# The Impact of Comorbidity on Severe Procedure-Coded Inpatient Events in Head and Neck Cancer and Thyroid Cancer Hospitalizations in Germany: A Nationwide DRG Analysis, 2005–2021

**DOI:** 10.3390/cancers18172860

**Published:** 2026-09-04

**Authors:** Lisa-Marie Müller-Anderski, Mussab Kouka, Peter Schlattmann, Orlando Guntinas-Lichius

**Affiliations:** 1 Department of Otorhinolaryngology, Jena University Hospital, D-07747 Jena, Germany; lisa.95145@web.de (L.-M.M.-A.); mussab.kouka@med.uni-jena.de (M.K.); 2 Department of Medical Statistics, Computer Sciences and Data Sciences, Jena University Hospital, D-07743 Jena, Germany; peter.schlattmann@med.uni-jena.de

**Keywords:** head and neck neoplasm, thyroid neoplasm, comorbidity, therapy, surgery, radiotherapy, chemotherapy, nationwide, procedure-coded inpatient events

## Abstract

Background: Comorbidity is an important determinant of treatment selection and in-hospital complications in patients with head and neck cancer (HNC) and thyroid cancer (TC), yet population-based evidence on this relationship remains limited. Methods: We analyzed nationwide Diagnosis-Related Groups (DRG) data from 1,552,028 inpatient HNC and TC treatments of patients aged ≥30 years in Germany between 2005 and 2021. The aim was to characterize the association of comorbidity and severe treatment-related procedure-coded inpatient events (IEs) with gender, age, tumor subsite, and treatment type. Results: The largest proportion of treatments occurred in patients aged 60–69 years (33.5%). The most frequent tumor subsites were the oropharynx, thyroid gland, oral cavity, larynx, and hypopharynx, with 35.52, 33.84, 33.60, 26.12, and 15.76 treatments per 100,000 population per year, respectively. Overall, 38% of cases had a Charlson Comorbidity Index (CCI) ≥ 1, with the highest mean CCI observed for C14 (other sites of the lip, oral cavity and pharynx) and C12 (piriform sinus). IEs requiring additional in-hospital treatment occurred in 19.3% of cases. After adjustment for age, tumor location, and treatment type, men had a lower risk of IEs than women (OR 0.929; CI 0.919–0.939; *p* < 0.001). Increasing comorbidity was associated with a higher IE risk, reaching a plateau at CCI ≥ 4 (OR 2.135; CI 2.029–2.246; *p* < 0.001). IE risk was high during chemotherapy/immunotherapy (OR 29.527; CI 28.897–30.170; *p* < 0.001), followed by surgery (OR 3.465; CI 3.433–3.498; *p* < 0.001), whereas radiotherapy showed the lowest risk (OR 1.339; CI 1.313–1.366; *p* < 0.001). Conclusions: These findings highlight substantial heterogeneity in comorbidity and IE risk among patients with HNC or TC.

## Simple Summary

This study examined Germany-wide hospital data from 1,552,028 treatments of head and neck cancer (HNC) and thyroid cancer (TC) between 2005 and 2021. The goal was to investigate, at the population level, how other health conditions affect the risk of procedure-coded inpatient events during HNC and TC treatment. More than one-third of treatments involved patients who had at least one additional serious health condition. Treatments for cancers of the hypopharynx and oropharynx had the highest burden of other illnesses. Severe procedure-coded inpatient events (IEs) requiring additional in-hospital therapy occurred during approximately 1 in 5 hospital treatments. Treatments of HNC or TC in patients with more health problems were significantly more likely to be associated with severe IEs. Women had a higher risk of IEs than men. The risk of IEs also differed by treatment type: chemotherapy/immunotherapy was associated with the highest risk, followed by surgery, while radiotherapy had the lowest risk. These findings highlight that a higher comorbidity burden demands greater attention to pre-treatment optimization, including prehabilitation, before initiating therapy.

## 1. Introduction

Patients with head and neck cancer (HNC) or thyroid cancer (TC) have a high prevalence of comorbidity [1,2]. Comorbidity is driven largely by the cancer risk factors tobacco use and alcohol consumption for HNC as well the aging population for HNC and TC [2,3]. Higher comorbidity is linked to treatment delay, deviation from treatment standards, reduced tolerance to therapy, higher risks of treatment-related complications, peri-treatment morbidity, and overall survival [4,5].

Treatment-related complications are manifold. Typical surgical complications are wound complications, fistula formation, bleeding, infections, airway issues, or flap failure infection [6]. Typical complications of radiotherapy are oral mucositis, pain, dysgeusia, salivary dysfunction, infections, or nutritional compromise [7]. Finally, typical complications of HNC or TC include chemotherapy includes mucositis, dysphagia, nephrotoxicity, hematologic toxicity, dehydration, or feeding tube dependence [8].

We are aware of only a few epidemiologic population-based studies on both comorbidity and treatment-related complications, as well as their interaction [4,9,10,11].

Since 2004, German hospitals have to submit a Diagnosis-Related Groups (DRG) coding to the insurance company of the patients to receive the reimbursement of hospital stays. In addition, the hospitals annually submit all hospitalization data to the Hospital Remuneration System (InEK) for a continual adjustment of the DRG system. The data are anonymized and also forwarded to the Federal Bureau of Statistics (DESTATIS; https://www.destatis.de/). The DRG data evaluated by the Federal Statistical Office includes various patient-related variables including comorbidity and treatment courses of all inpatients who were discharged in virtually all German hospitals during the reporting year and can be used for scientific analyses [12,13]. The DRG data do not allow direct identification of clinically adjudicated in-hospital complications. They do, however, allow identification of procedure-coded inpatient events occurring in addition to the primary treatment and associated with additional diagnosis coding. For instance, this data source was used to analyze the nationwide in-hospital comorbidity and indirect indicators of treatment-related complications following colonic cancer resection from 2012 to 2015 [14]. Previously, we used DRG data to report that treatment patterns had changed for nearly all subsites and therapy types of HNC in Germany with relevant gender disparities between 2005 and 2018 [15]. In-hospital mortality had not changed over time [16]. Moreover, we recently demonstrated that older age cohorts (≥65 to <80 years and ≥80 years) were mainly responsible for the increase in treatment rates for surgery, radiotherapy, and chemotherapy/immunotherapy, while younger cohorts (≥35 to <65 years) showed decreasing treatment rates [17].

Now we used the DRG data from 2005 to 2021 to analyze the nationwide comorbidity of patients with HNC and TC requiring inpatient treatment and severe procedure-coded inpatient events in Germany, focusing on their interaction and the influence of gender, age, tumor subsite, and treatment types.

## 2. Methods

### 2.1. Data Source

Data of individual inpatients treated from January 2005 to December 2021 (17 years) were obtained from the nationwide German DRG statistics. The authors used anonymized data supplied by the German Federal Bureau of Statistics (DESTATIS). The anonymization of such data is regulated in § 16 Bundesstatistikgesetz (German Federal Statistics Act). The datasets are reviewed for confidentiality, and results involving fewer than three cases are blocked and not released. Consequently, no results are released that could allow conclusions to be drawn about individual cases. In this constellation, ethics approval from the local ethics committee was not needed. It is important to note that the datasets include inpatient treatments and not individual patients (i.e., the number of treatments is higher than the number of patients). Therefore, in this analysis, we consistently refer to “treatments” rather than “patients”. By definition of the DRG system, if a discharge and re-admission occurs within 30 days, the two inpatient treatments are grouped together into a single case.

### 2.2. Patient Cohort Selection

All database queries regarding case numbers at DESTATIS were performed using SAS version 9.4 (SAS Institute, Cary, NC, USA). Since the queries and case counts were subject to confidentiality restrictions, the queries were created using a truncated sample database and sent via email to DESTATIS. The case counts were queried in an iterative process using various characteristic values; that is, the relationships between patient characteristics, treatment, and inpatient events were analyzed sequentially in separate queries. Separate queries with different combinations of variables were required. It was not possible to represent all the relationships between the parameters of interest to be analyzed in a single query. This would have resulted in subgroups that were too small. Also because of the confidentiality statutes, it is by law not allowed to issue such small subgroups. This would lead to the data being blocked, meaning the analyses would not be made available. Queries with ICD codes up to the second or third decimal place also had the same problem: they also resulted in subgroups that were too small. The repeated queries using different combinations of parameters resulted in data files of varying sizes, meaning that the number of cases considered differed across the individual queries. This also explains, for example, why the gender ratios vary across the queries. Because of the confidentiality restrictions applying to the DESTATIS data, several separate queries with different combinations of variables were required. Consequently, the number of treatment episodes and the distribution of individual variables may differ between query outputs. The specific query populations used for the descriptive and regression analyses are summarized in Supplementary Table S5. The ordinal regression model was calculated by DESTATIS using 1,552,028 observations, of which 1,551,955 were included in the final model; 73 observations were excluded because of missing sex information.

The queried variable values were encoded as follows. The hospitalizations with a primary diagnosis for HNC and TC of the International Classification of Diseases, 10th Edition, German Modification (ICD-10-GM) were analyzed: C01-C14, C30-C33, and C73 (details in Supplementary Table S1). Subsequently, all cases were grouped according to the operation and procedure code (OPS, version 2005 to 2021; details in Supplementary Table S2). The treatments administered were categorized into surgery, radiation therapy, cytostatic chemotherapy/immunotherapy. The variables were numerically coded and recorded using a dichotomous classification of “yes” and “no.” In a next data query, the variable gender (male, female) was included in the query. In the last step, the variable age cohorts were included in the query. In the dataset, age was grouped into cohorts of 30–39, 40–49, 50–59, 60–69, 70–79, 80–89, 90–99, and 100–109 years. For each inpatient treatment, the age cohort was known, but not the exact age. There was one exception: For the multivariable analysis, it was possible to include the patients’ exact age in years. The age cohorts from 0 to 29 years and 100 to 109 years were excluded from some calculations, because too many subgroups with <3 patients were generated. Therapy analyses were only possible for the age cohorts older than 39 years of age due to the same reasons. Because of the confidentiality statutes, it is by law not allowed to issue such small subgroups. This was the reason, why the number of inpatient treatments could vary between different calculations. Clinical variables (e.g., TNM classification, tumor histology) were not available, as these are not billing-relevant.

### 2.3. Definition of Comorbidity

Comorbidity refers to the simultaneous presence of one or more diagnostically distinct clinical conditions in addition to a primary underlying disease [18]. According to German coding guidelines, secondary diagnoses include diseases or conditions that either coexist with the primary diagnosis or develop during the hospital stay [19,20]. In addition to the primary diagnosis, DRG statistics can also record up to 89 secondary diagnoses per patient treatment. These are reported in DRG statistics using ICD codes via the variables “icd_nd1–icd_nd89.” The CCI components were identified using predefined ICD-10 code sets, with hierarchical assignment applied where a diagnosis could meet criteria for more than one severity category. The detailed code definitions and hierarchy are provided in Supplementary Table S3. Because the study used administrative data, the CCI represents coded comorbidity rather than clinically adjudicated comorbidity. In particular, distinctions between severity categories depend on the specificity and completeness of the ICD-10 coding. In this study, 19 relevant comorbidities (details in Supplementary Table S3) were considered to define the comorbidity according to the Charlson Comorbidity Index (CCI) [21,22]. The CCI is used in oncology as a standardized tool for assessing mortality risk. To this end, 19 possible diseases are recorded and evaluated. To determine the severity of comorbidity, fixed point values (1, 2, 3, 6) are assigned to this disease. The point values for the individual disease present are then added together. If no comorbidities are present, the value is 0. The primary cancer being studied is excluded from the CCI value.

### 2.4. Definition of Severe Procedure-Coded Inpatient Events

The OPS codes are used to code any surgeries and other medical procedures in inpatient care and in the outpatient surgery sector [23]. The respective OPS codes for resource-intensive procedures, called in this analysis inpatient events (IEs), potentially indicating complications, are recorded in the DRG statistics using the variables “ops_ko1-ops_ko101.” Resource-intensive procedures or IEs associated with the primary treatment of HNC and TC during inpatient care can be analyzed indirectly by drawing conclusions from the documented treatment measures [23,24]. For example, based on the treatment measure “transfusion of blood cells,” it can be assumed that bleeding occurred as an IE, i.e., as a complication. The OPS-code selection for the IE selection is summarized in Supplementary Table S4. OPS codes related to the therapy itself were removed from the compilation. OPS codes were counted only if additional ICD codes were coded at discharge (case closure) as secondary diagnoses that had not yet been coded at admission. The aim was to distinguish, as far as possible, cases in which an event (e.g., bleeding) was not the reason for admission but occurred only during inpatient treatment as inpatient event. An overview of the number of cases per data file, depending on the specific query and parameter configuration, is summarized in Supplementary Table S5 and Figure S1.

### 2.5. Statistical Analysis

The population of Germany of the years 2005–2021 was used to calculate the treatment rates (incidence rates). Population data were provided by the German Federal Bureau of Statistics (DESTATIS). The average German population between 2005 and 2021 was 82,083,021.5 residents. The average population ≥30 years and ≥40 years was 56,987,938.47 residents and 46,619,982.47 residents, respectively.

Descriptive statistical analyses were performed using IBM SPSS version 27.0 statistical software for Windows (Armonk, NY, USA). Mean values ± standard deviation are presented if not otherwise indicated. All other calculations were carried out using SAS version 9.4 (SAS Institute, Cary, NC, USA). For descriptive purposes, treatment episodes with at least one event were assigned to a mutually exclusive hierarchical event category, with each treatment episode represented once. To investigate potential factors influencing the occurrence of coded inpatient events, an ordinal regression analysis was conducted. For the ordinal regression analysis, the number of qualifying procedure-coded inpatient events identified for each treatment episode was used as the dependent variable and categorized as 0, 1, 2, 3, or ≥4 events. The model was used to perform a multivariable analysis of clinical and demographic predictors in relation to the occurrence of procedure-coded inpatient events as the total number of events per case. The model considered the same set of variables, i.e., age (only here metric, in years), and gender (dichotomous), tumor location (based on primary diagnosis ICD code), and types of treatment (surgery [yes/no], radiotherapy [yes/no], and chemotherapy/immunotherapy [yes/no]). F-statistics were used to examine the significance of each partial effect, that is, the significance of a variable with all the other variables in the model. The GLIMMIX procedure in the SAS statistical software was used to estimate the parameters of the ordinal regression models. The models are based on a cumulative logit link, assuming that the transition probabilities between the ordinal categories follow a logistic distribution. Parameter estimation was performed using the maximum likelihood method with the Newton–Raphson algorithm as the optimization method. Odds ratio (OR) and 95% confidence intervals (CI) are presented to describe the association between variables and extent of comorbidity and the occurrence of inpatient events, respectively. In addition, intercepts (threshold parameters or cut points) were estimated in the model, which defined the limits between the ordinal categories of the dependent variable on a latent continuous scale and thus form the basis for the calculation of the categorical probabilities. For all statistical tests, significance was two-sided and set to *p* < 0.05.

## 3. Results

### 3.1. The Population of Treatment Episodes with Head and Neck Cancer and Thyroid Cancer and ≥30 Years of Age Treated as Inpatients in German Hospitals Between 2005 and 2021

A total of 1,552,028 inpatient treatment episodes were performed in the 17-year period resulting on average in 91,296 inpatient treatments per year for patients with HNC or TC ≥ 30 years of age. Age and sex information was available for 1,228,215 treatment episodes in the sex-stratified DESTATIS query. The largest number of cases was among patients aged 60 to 69 years (33.5%), follow by patients aged 50 to 59 years (29.7%). Out of the 1,552,028 inpatient treatments, 746,446 treatments (48.0%) were performed on male patients and 481,769 (31.0%) on female patients. Sex was not available in the sex-stratified query for 323,813 treatment episodes (20.9% of the overall cohort). When excluding the patients with thyroid cancer, the balance shifts even more clearly in favor of men: excluding thyroid cancer, 667,185 male patients (54.5%) and 325,877 female patients (26.6%) were treated (gender unknown for 231,080 treatments; 18.9%). An overview about the tumor subsites is given in Table 1. The ICD codes per gender and age cohort are shown in detail in Supplementary Table S6 and Figure S2. On the level of the ICD codings, the most frequent codings were C73 (thyroid cancer), larynx (C32), oropharynx (C10), and hypopharynx (C13) with 33.84, 26.12, 17.06, and 14.72 treatments per 100,000 population per year, respectively. When cumulating the codings to TNM subsites, the most frequent TNM tumor subsites were oropharynx (C01, 09–10, 14), thyroid gland (C73), oral cavity (C00, 02–06), larynx (C32), and hypopharynx (C12–13) with 35.52, 33.84, 33.60, 26.12, and 15.76 treatments per 100,000 population per year, respectively.

### 3.2. Comorbidity

**Figure 1:**
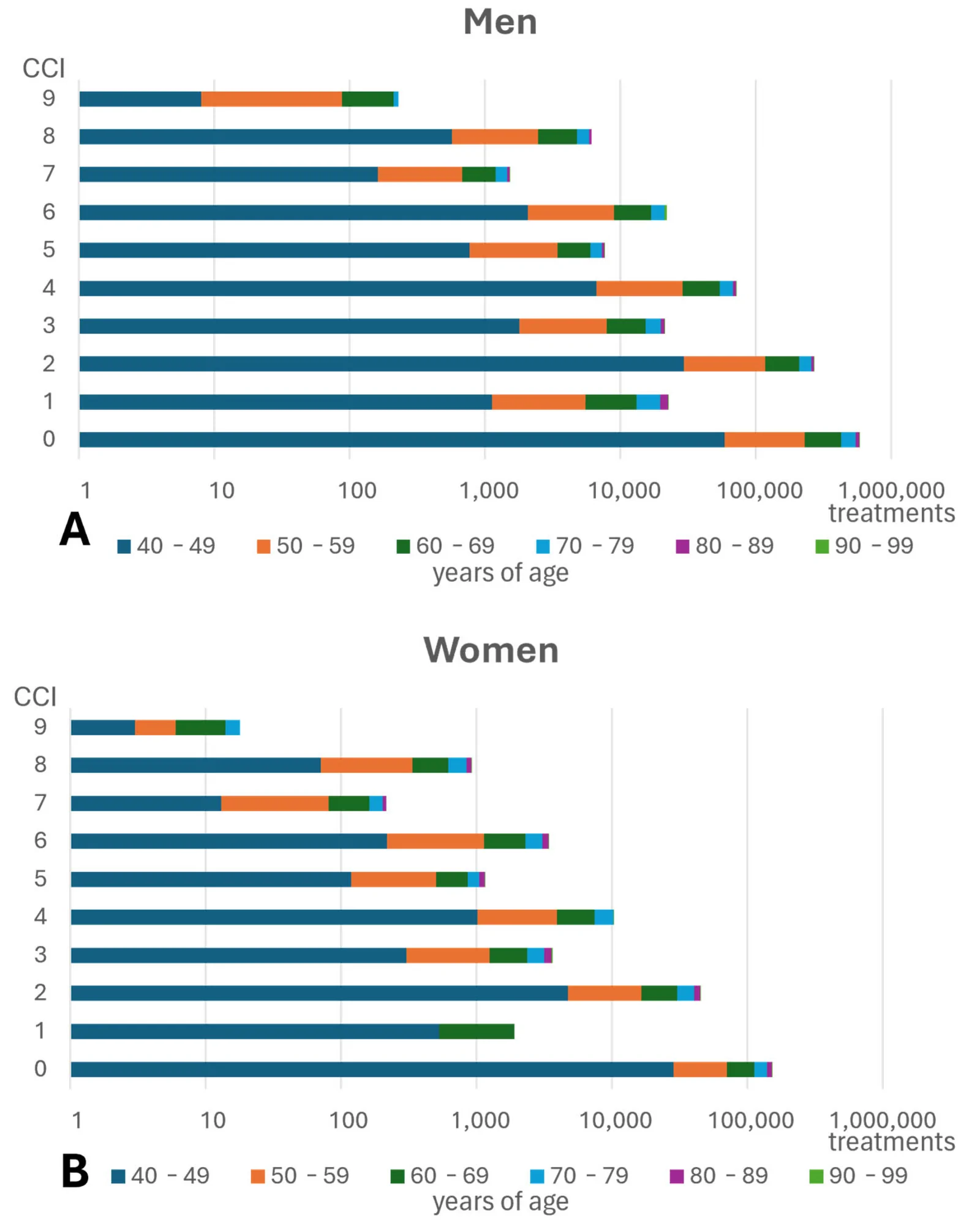
Comorbidity severity using the Charlson Comorbidity Index (CCI) in different age cohorts (color-coded) per absolute number of in-hospital treatments in head and neck cancer (HNC) and thyroid cancer (TC) cases between 2005 and 2021 in German hospitals. (**A**) Men, (**B**) women. X-axis: Absolute number of treatments, logarithmic scale; Y-axis: Charlson Comorbidity Index (CCI).

A total of 961,824 out of the 1,552,028 inpatient cases (61.97%) were patients without comorbidity due to the CCI (Table 2). Regarding having a comorbidity, the most frequent classes were CCI 2, CCI 4, and CCI 1 with 376,539 cases (24.26%), 97,412 cases (6.28%), and 35,370 cases (2.28%), respectively. The average CCI was 1.03. Median CCI was 0. Gender data in the CCI dataset were available for 1,222,367 treatments, i.e., for 78.8% of the entire 1,552,028 inpatient cases. In this subset of 1,222,367 treatments, women had no comorbidity more often (69.29%) compared to men (57.77%). The CCI per gender and age cohort is shown in Figure 1. Highest mean CCIs were seen in patients with cancer of other sites in the lip, oral cavity and pharynx (ICD: C14; 1.466), followed by piriform sinus cancer (C12; 1.462), hypopharyngeal cancer (C13; 1.458) and tonsil cancer (1.394) (Supplementary Table S7). The lowest mean CCIs were seen in patients with nasal cavity/middle ear (0.704) and thyroid gland (0.514) cancer.

### 3.3. Therapy

Therapy details according to OPS codes of 572,017 treatments (out of the 1,552,028 inpatient cases; 36.97%) were available for treated cases with HNC or TC and ≥40 years of age, and are presented in Table 3, Supplementary Table S8 and Figure S3. Surgery as monotherapy was the dominating therapy type (79.6% of all cases), followed by radiotherapy (11.3%), and chemotherapy/immunotherapy (7.3%) for women and men. In men, the highest incidence rates (treatment rates) were seen for age cohorts for 60- to 69-year-old patients with the maximum for surgery at 125.4 per 100,000 population. The only exception was surgery combined with radiochemotherapy. Here, the highest treatment rate was seen for 70- to 79-year-old male patients. For women, the highest incidences were also seen for 60- to 69-year-old patients with the maximum for surgery at 48.2 per 100,000 population. The exceptions to this were the results regarding the radiochemotherapy. Here, 70- to 79-year-old female patients showed the highest incidence rate. For surgery and radiochemotherapy, the highest treatment incidence was observed for 50–59-year-old female patients.

### 3.4. Procedure-Coded Inpatient Events

**Figure 2:**
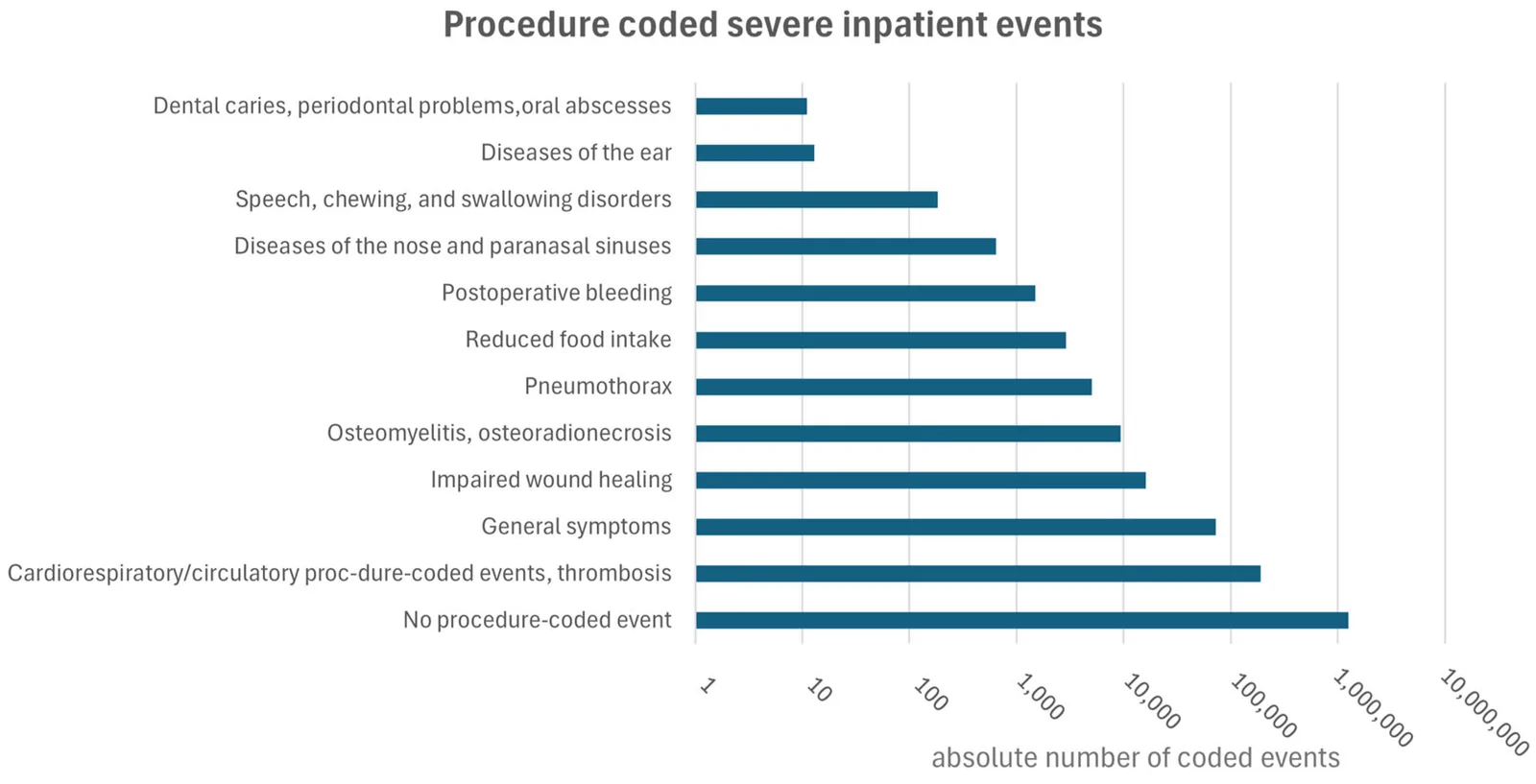
Procedure-coded inpatient events needing additional in-hospital treatment of head and neck cancer (HNC) and thyroid cancer (TC) cases treated between 2005 and 2021 in German hospitals. X-axis: Absolute number of treatments, logarithmic scale.

No Ies, under the OPS-based definition were observed in 1,252,942 inpatient treatments (80.73%). IEs occurred in 299,086 cases (19.27%). All IEs are listed in Table 4 and shown in Figure 2. The most important inpatient IEs were myocardial infarction, respiratory arrest, circulatory IEs (190,355 cases; 63.65%), general symptoms (72,787 cases; 24.34%), and impaired wound healing (16,195 cases; 5.41%).

### 3.5. Factors Associated with Procedure-Coded Inpatient Events

The inpatient event model included the independent variables age, gender, comorbidity, tumor location, and the treatment modalities. The F-statistics showed that all variables included in the model showed an independent and highly significant association to the occurrence of IEs (age, F = 162, *p* < 0.001; gender, F = 186, *p* < 0.001; comorbidity, F = 2523, *p* < 0.001; subsite F = 1784, *p* < 0.001; surgery, F = 66,584, *p* < 0.0001; radiotherapy, F = 831, *p* < 0.001; chemotherapy/immunotherapy, F = 94,335, *p* < 0.001). Table 5 shows the results of the ordinal logistic regression model for therapy related inpatient events. The intercepts in the model correspond to the transitions between the number of events (e.g., 1, 2, 3 or more events). As age increases, a higher probability for events was observed (OR = 1.003, CI = 1.002–1.003; *p* < 0.001). Men, on average, had fewer events than women (OR = 0.929; CI = 0.919–0.939; *p* < 0.001), given the same age, tumor location, and comparable treatment. With regard to tumor subsites, more events were observed for all locations compared to the reference category C73 (thyroid cancer), indicating a greater prevalence of events in all other tumor regions. Particularly pronounced effects were observed for C03 (gum; OR = 5.749; CI = 5.584–5.920; *p* < 0.001), C33 (trachea; OR = 4.953; CI = 4.650–5.276; *p* < 0.001), C04 (floor of the mouth; OR = 4.677; CI = 4.583–4.774; *p* < 0.001), C06 (unspecified lip, mouth, pharynx (C14); OR = 4.450;CI = 4.312–4.593; *p* < 0.001), and C02 (non-specified parts of the tongue; OR = 4.002; CI = 3.915–4.091; *p* < 0.001), suggesting a particularly strong association between these sites and an increased occurrence of inpatient events. Furthermore, a higher CCI was associated with more events (OR = 2.135; *p* < 0.001). With regard to treatment variables, chemotherapy/immunotherapy showed the strongest association with procedure-coded inpatient events (OR = 29.527; CI = 28.897–30.170; *p* < 0.001) followed by surgery (OR = 3.465; CI = 3.433–3.498; *p* < 0.001). The probability was lowest for radiotherapy (OR = 1.339; CI = 1.313–1.366; *p* < 0.001).

## 4. Discussion

Patients with HNC and TC in Germany represent—as in other countries—a heterogeneous population with a considerable burden of comorbidity. Comorbid conditions play a key role in both treatment selection and the risk of in-hospital procedure-coded events as indirect indication of the occurrence of complications. The present population-based study of 1,552,028 inpatient treatments of HNC and TC patients over a period of 17 years (i.e., the present study is based on about 91,296 treatments per year) showed that the likelihood of inpatient events varies depending on patient characteristics such as age, sex, comorbidity, tumor site, and type of therapy. Overall, more complex treatment modalities and higher comorbidity are associated with an increase in procedure-coded inpatient events requiring additional in-hospital treatment.

Comorbidity alone is not precise enough as predictor. It is important to take into account the severity of each other comorbid condition. Therefore, it is important not only to use a standardized assessment tool, but also to choose a method that takes into account the severity of each condition. That is a key advantage of the CCI [25,26]. A total of 38% of the cases had a CCI ≥ 1. It has been demonstrated on multiple occasions that ICD coding derived from DRG data is suitable for calculating the CCI [13,22,27]. Nevertheless, since the ICD coding of additional conditions is relevant to revenue only in certain cases, it is likely that additional conditions are often not fully coded. Therefore, it is more likely that the prevalence of relevant comorbidity was underestimated in the present study. Studies frequently report that around one-third to over half of patients present with at least one comorbid condition, largely driven by tobacco use and alcohol consumption [9,28,29,30]. There are a few population-based studies confirming this high prevalence of comorbidity using standardized assessment tools: a CCI ≥ 1 was reported for 48% in a cohort from Thuringia, Germany, from 2009 to 2011 [3]. In the Belgian Cancer Registry, a CCI ≥ 1 was found in 39% of the 9245 HNC patients registered between 2009 and 2014 [4]. In the Danish DAHANCA cancer registry, a CCI ≥ 1 was reported in 46% of the 9388 HNC patients planned for radiotherapy from 1992 to 2008 [31]. Hence, the prevalence for Germany as a whole appears broadly comparable to that reported in other HNC cohorts, although direct comparisons are limited by differences in study populations and methods of comorbidity ascertainment. The CCI distribution and its association with procedure-coded inpatient events should nevertheless be interpreted cautiously, because the CCI in the present study represents coded rather than clinically adjudicated comorbidity. It appears that comorbidity has not decreased. Smoking and alcohol are not only major risk factors for HNC, but also for many other conditions, like, for instance, cardiovascular diseases, that are relevant to comorbidity [32,33]. Indirectly, the results suggest that campaigns to encourage people to quit smoking and drinking have likely had little success, at least among the HNC cohort studied here. In any case, the CCI was associated with the occurrence of severe inpatient events in the present analysis. This finding indicates a population-level association between coded comorbidity burden and additional inpatient management, but does not establish that the CCI independently predicts clinically defined complications or that it can directly be used for individual treatment planning.

Principally, using ICD codes in DRG data allows the indirect identification of severe inpatient complications by evaluating secondary diagnoses and procedure codes within administrative claims. Unfortunately, the DRG coding does not allow for direct and unambiguous identification of complications. Therefore, like others before us, we had to take the indirect route via the IEs. The analysis had to be limited to a selection of the most serious procedure-coded events. It can therefore be assumed that the incidence of serious complications was likely underestimated. However, it was not possible to examine disease-specific or treatment-specific complications in detail, first because ICD coding does not clearly reflect them, and second because this would have resulted in a vast number of subgroups of coded inpatient events. Third, routine administrative data often miss complications unless they are severe enough to require additional surgeries, transfusions, or prolonged hospital stays [19,34,35]. Fourth, the potential for complications varies greatly between different surgeries—or even when comparing surgery, radiotherapy, and chemo-/immunotherapy. A generic approach for grading at least surgical complications independently of the type and field of surgery including head and neck surgery is the Clavien–Dindo classification (CDC) [36,37]. The Clavien–Dindo classification classifies the severity of a complication based on the type of therapy required to treat the complication. Unfortunately, there is currently no standard for calculating the CDC from DRG data [38,39]. We therefore limited our analysis to severe general inpatient events, extracted from the OPS codes, as others have done before [40,41]. Hence, the aim was not to analyze risk factors for specific complications. The aim was to identify factors generally associated with procedure-coded inpatient events requiring additional in-hospital therapy, which may serve as indirect indicators of potentially severe complications for HNC and TC patients. Cardiovascular events were frequent in Germany in HNC patients. This is in line with other publications from other countries [33,42,43,44,45]. Identifiable risk factors include increasing age and comorbidity [43]. More difficult to explain in our study is the finding that women had a higher risk of severe inpatient events after adjusting for other relevant factors. The data available is inconsistent. Sometimes gender effects are described in one direction or the other; sometimes they are not [16,46,47,48]. Men were less likely to experience an IE in the present study, but the gender effect was clinically very small (OR 0.929) in relation to the large dataset (>1.2 million treatments). In any, case, our methodological approach did not allow for a causal analysis of the gender effect. This effect should not be interpreted biologically as this would need a careful stratification by tumor site, particularly thyroid cancer, which was not possible with the DRG-based dataset.

The overall observed inpatient procedure-coded event rate was 19.27%. This figure should not be interpreted as a clinical complication rate, because the primary endpoint was derived indirectly from administrative procedure and diagnosis codes. There are only a few population-based studies on complications associated with the treatment of HNC or TC. For 3001 geriatric HNC patients undergoing surgery, i.e., a population with high comorbidity, a complication rate of 27.5% is reported in the U.S. Vizient Clinical Data Base during 2009 and 2012 [49]. Most often, selected specific complications are addressed: the 2009–2011 U.S. Nationwide Inpatient Sample (NIS) revealed a 4.3% complication rate and a 4.3% transfusion rate [50]. According to the U.S. SEER cancer registry, 86% of 1502 HNC patients aged ≥ 66 years treated by radiotherapy or radiochemotherapy were hospitalized for acute toxicity [10]. DAHANCA analyzed acute toxicities in proton versus photon therapy for HNC in Denmark in a prospective trial [51]. Strictly speaking, this was therefore not a population-based study. The population-based situation is even less clear for modern immunotherapy. The Japanese Ministry of Health, Labor and Welfare requested the manufacturer to conduct post-marketing surveillance of all 607 cases of nivolumab treatment for HNC in Japan between 2017 and 2020. The data showed that the most common severe adverse events were interstitial lung disease (1.2%), diarrhea (0.8%), and hepatic dysfunction (0.7%) [52]. However, the data do not allow for a distinction between acute complications and long-term adverse events. For TC, the data situation is no better. There is one population-based assessment of complications following surgery for TC using SEER data from 1998 to 2011 [53]. Of the 27,912 patients 6.5% developed general postoperative complications and 12.3% thyroid surgery-specific complications. Age and CCI were important risk factors. An analysis of the NIS database from 1998 to 2008 revealed that 5.5% of 119,567 patients who underwent thyroidectomy for TC developed hypocalcemia [54]. These examples show that comprehensive population-based data on complications associated with the treatment of HNC and TC have been insufficient to date, and that the methods used to collect data on these complications vary widely.

The most severe inpatient complication is be treatment-related death. This was not considered in the present study, as we had already examined it in detail recently [15]. Analyzing DRG data in the same setting from 2005 to 2018, we found an average in-hospital mortality of 3.9%. Main risk factors were age ≥ 65 years and palliative treatment [16].

DRG data are collected prospectively through the routine hospital coding process from virtually all German hospitals. Nevertheless, this methodology has several limitations. First, comparing DRG coding with actual patient records can reveal discrepancies, as billing codes are optimized for reimbursement rather than clinical research [55]. Not all comorbidities are relevant for the reimbursement. This could have led to a selection bias in the reporting of comorbidities and may be responsible for the unusual CCI distribution with relatively high rates for CCI 2 and CCI 4, while CCI 1 and CCI 3 were less frequent. This distribution may partly reflect the structure of the ICD-10 coding algorithm used in administrative data. In addition, differences in coding intensity related to reimbursement and documentation practices may have contributed. Hence, the CCI assessment represents another limitation of the study. Although the CCI is widely used for risk adjustment in administrative data, its validity depends on the underlying ICD-10 coding and on the specificity with which individual comorbid conditions are documented. In our dataset, some CCI categories may not have been distinguishable with the same clinical granularity as in validated fourth-digit ICD-10 algorithms. In addition, the metastatic solid tumor component may overlap with regional lymph-node disease in HNC, potentially reflecting tumor stage rather than independent comorbidity. Consequently, the CCI distribution and its association with procedure-coded inpatient events should be interpreted cautiously.

Second, the reasons for therapy decisions remain unclear. Specific reasons for perioperative complications can also remain unclear [56]. It has to be emphasized that all conclusions were drawn at the level of the hospital stay rather than at the patient-level. For data protection reasons, highly granular queries using ICD or OPS codes were only possible to a limited extent, down to the decimal places. Together with the uncertain validity of the complication proxies, complication timing, and a coding-intensity bias, this leads to significant uncertainties regarding the recording of the actual complication based on DRG statistics. The DRG data do not allow a causal association with underlying reasons. Reverse causation between comorbidity and complications and confounding by stage and treatment indication could have occurred. This is also important when interpreting the impact of the therapy type on inpatient events: the strong association between chemotherapy/immunotherapy and occurrence of IEs should not be interpreted as a direct causal effect of chemotherapy/immunotherapy, because treatment intensity and procedure coding may be closely linked in administrative data. In particular, the very large odds ratio observed for chemotherapy/immunotherapy should be interpreted as an association with the corresponding treatment episode and additional procedure-coded inpatient management, rather than as evidence of a causal increase in complication risk. Third, anonymized DRG data do not allow a linkage to other patient and treatment specific data. Most importantly, TNM staging data, histopathology data, and survival information are not available. It is well known that comorbidity affects treatment selection and overall survival [9,30,57]. We would like to point out once again that we were only able to examine the inpatient sector. The treatment figures for nonsurgical therapy suggest that a significant proportion of nonsurgical treatment for HNC is provided on an outpatient basis. The potential complications that may arise in that setting are not considered. Finally, a further limitation concerns the cumulative logit model used for the multivariate ordinal outcome analysis. This model assumes proportional odds, i.e., that the association between each covariate and the outcome is constant across the cumulative thresholds. This assumption was not formally assessed in the present analysis. Consequently, the reported odds ratios should be interpreted as common effects across the cumulative thresholds rather than as threshold-specific estimates. Given the very large sample size, even relatively small departures from proportionality may be statistically relevant, and the possibility of threshold-specific associations cannot be excluded. The estimates should therefore be interpreted in the context of this modeling assumption.

Machine learning methods are increasingly being used to make predictions based on HNC registry data [58,59,60]. Related to DRG data, machine learning has so far only been used for DRG grouping (for example, [61]). We were unable to identify any work that used DRG data from HNC patients in combination with machine learning tools for predicting factors associated with comorbidity and in-hospital events as indirect indications for complications. This would be a sensible next step in conducting a more in-depth data analysis. A REporting of studies Conducted using Observational Routinely-collected Data (RECORD) statement on the use routinely-collected health data is presented in Supplementary Table S9.

## 5. Conclusions

In this nationwide analysis of inpatient treatment episodes for HNC and TC in Germany, higher comorbidity was associated with a higher frequency of procedure-coded inpatient events. The findings indicate a population-level association between coded comorbidity burden and additional inpatient management during cancer treatment. However, because the outcome was derived from administrative procedure and diagnosis codes rather than clinical adjudication, the results should not be interpreted as direct estimates of individual complication risk or as evidence of causality.

## Figures and Tables

**Table 1:** Overview about the analyzed head and neck cancer and thyroid cancer inpatient treatments between 2005 and 2021 for patients ≥ 30 years of age (result of the search in the DESTATIS database: *N* = 1,552,028 treatments for patients ≥ 30 years of age).

ICD	Number of Treatments, Absolute *N*	Number of Treatments, Relative %	Average Number of Treatments per Year, Absolute *N*	Average Number of Treatments per 100,000 Population per Year (Incidence Rate) *	Tumor Subsite
all	1,552,028	100	91,295.76	160.20	-
C73	327,886	21.13	19,287.41	33.84	Thyroid gland
C32	253,082	16.31	14,887.18	26.12	Larynx
C10	165,253	10.65	9720.76	17.06	Oropharynx
C13	142,589	9.19	8387.59	14.72	Hypopharynx
C04	115,375	7.43	6786.76	11.91	Floor of the mouth
C09	94,305	6.08	5547.35	9.73	Tonsil
C02	91,896	5.92	5405.65	9.49	Unspecified part of the tongue
C01	75,641	4.87	4449.47	7.81	Base of the tongue
C07	35,344	2.28	2079.06	3.65	Parotid gland
C11	34,692	2.24	2040.71	3.58	Nasopharynx
C31	32,149	2.07	1891.12	3.32	Paranasal sinus
C05	31,829	2.05	1872.29	3.29	Palate
C03	31,103	2.0	1829.59	3.21	Gums
C30	29,783	1.92	1751.94	3.07	Nasal cavity and middle ear
C06	27,883	1.8	1640.18	2.88	Parts of the mouth
C00	27,471	1.77	1615.94	2.84	Lip
C08	10,194	0.66	599.65	1.05	Unspecified major salivary gland
C12	10,084	0.65	593.18	1.04	Piriform sinus
C14	8904	0.57	523.76	0.92	Other sites in the lip, oral cavity and pharynx
C33	6565	0.42	386.18	0.68	Trachea
ICD codes cumulated to tumor subsites					
C01, 09–10, 14	344,103	22.17	20,241.35	35.52	Oropharynx
C00, 02–06	325,557	20.98	19,150.41	33.60	Oral cavity
C12–13	152,673	9.84	8980.76	15.76	Hypopharynx
C07-08	45,538	2.93	2678.71	4.70	Salivary glands

* related to an average population of 56,987,938.47 residents ≥ 30 years.

**Table 2:** Charlson Comorbidity Index (CCI) distribution in the study cohort, 2005–2021 (result of the search in the DESTATIS database: *N* = 1,552,028 treatments for patients ≥ 30 years of age; when including gender as an additional parameter, the the search in the DESTATIS database yielded: *N* = 1,222,367 treatments for patients ≥ 30 years of age). The sex-specific values in this table are derived from the separate CCI + sex query comprising 1,222,367 treatment episodes (see Supplementary Table S5) and therefore do not correspond to the full cohort of 1,552,028 treatment episodes.

	All		Men		Women	
CCI	Number, Absolute *N*	Number, Relative %	Number, Absolute *N*	Number, Relative %	Number, Absolute *N*	Number, Relative %
All treatments *	1,552,028	100	1,002,705	100	219,662	100
0	961,824	61.97	579,221	57.77	152,200	69.29
1	35,370	2.28	22,669	2.26	1910	0.87
2	376,539	24.26	269,105	26.84	45,525	20.73
3	28,948	1.87	21,154	2.11	3658	1.67
4	97,412	6.28	71,695	7.15	10,412	4.74
5	10,084	0.65	7603	0.76	1166	0.53
6	29,586	1.91	22,057	2.20	3435	1.56
7	1967	0.13	1516	0.15	216	0.10
8	8117	0.52	6091	0.61	920	0.42
9	323	0.02	228	0.02	18	0.01
10	1824	0.12	1366	0.14	202	0.09
11	26	<0.01	NA	NA	NA	NA
12	8	<0.01	NA	NA	NA	NA

* the sum of men and women is not the same as the total number, as the gender was not given for all subgroups of treatment cases. NA = not applicable, because cells with fewer than three treatment episodes are suppressed in accordance with DESTATIS confidentiality requirements.

**Table 3:** Type of inpatient therapy for head and neck cancer and thyroid cancer 2005–2021 for patients ≥ 40 years of age (result of the search in the DESTATIS database: *N* = 572,017 treatments for patients ≥ 40 years of age). The therapy-specific values in this table are derived from the separate therapy query comprising 572,017 treatment episodes (see Supplementary Table S5) and therefore do not correspond to the full cohort of 1,552,028 treatment episodes.

	Surgery			Radiotherapy			Chemotherapy/Immunotherapy			Radiochemotherapy			Surgery and Chemotherapy			Surgery and Radiotherapy			Surgery and Radiochemotherapy		
Age, Cohorts in Years	Abs., *N*	Rel., %	IR	Abs., *N*	Rel., %	IR	Abs., *N*	Rel., %	IR	Abs., *N*	Rel., %	IR	Abs., *N*	Rel., %	IR	Abs., *N*	Rel., %	IR	Abs., *N*	Rel., %	IR
**Men**																					
All (40+)	303,831	100	80.7	49,649	100	13.2	33,569	100	8.9	1758	100	0.5	719	100	0.2	5311	100	1.4	80	100	<0.1
40–49	30,898	10.17	29.5	4892	9.85	4.7	2654	7.91	2.5	136	7.74	0.1	66	9.18	0.1	459	8.64	0.4	13	16.25	<0.1
50–59	87,227	28.71	83.5	15,969	32.16	15.3	10,602	31.58	10.1	479	27.25	0.5	260	36.16	0.2	1522	28.66	1.5	15	18.75	<0.1
60–69	100,157	32.96	125.4	17,265	34.77	21.6	12,611	37.57	15.8	570	32.42	0.7	244	33.94	0.3	1831	34.48	2.3	23	28.75	<0.1
70–79	65,338	21.50	109.3	9228	18.59	15.4	6384	19.02	10.7	436	24.80	0.7	124	17.25	0.2	1166	21.95	2.0	24	30.00	<0.1
80–89 *	18,917	6.23	73.5	2160	4.35	8.3	1285	3.83	4.8	137	7.79	0.5	25	3.48	0.1	316	5.95	1.2	5	6.25	<0.1
90–99 *	1294	0.43		135	0.27		33	0.10		-	-		-	-		17	0.32		-	-	
**Women**																					
All (40+)	151,515	100	36.4	14,892	100	3.6	8331	100	2.0	480	100	0.1	187	100	<0.1	1668	100	0.4	27	100	<0.1
40–49	26,001	17.16	25.5	1232	8.27	1.2	741	8.89	0.7	35	7.29	0.0	18	9.63	<0.1	125	7.49	0.1	-	-	-
50–59	40,614	26.81	39.0	4030	27.06	3.9	2257	27.09	2.2	105	21.88	0.1	56	29.95	0.1	394	23.62	0.4	11	40.74	<0.1
60–69	40,978	27.05	48.2	4616	31.00	5.4	2732	32.79	3.2	140	29.17	0.2	62	33.16	0.1	497	29.80	0.6	4	14.81	<0.1
70–79	29,620	19.55	40.7	3357	22.54	4.6	1856	22.28	2.5	142	29.58	0.2	32	17.11	<0.1	427	25.60	0.6	9	33.33	<0.1
80–89 *	12,814	8.46	27.6	1476	9.91	3.2	723	8.68	1.4	58	12.08	0.1	19	10.16	<0.1	213	12.77	0.4	3	11.11	<0.1
90–99 *	1488	0.98		181	1.22		22	0.26		-	-		-	-		12	0.72		-	-	

* the age cohorts of the German population data do not differentiate between both genders; IR = incidence rate per age cohort per 100,000 population related to the German population 40+ years of age; abs. = absolute; rel. = relative.

**Table 4:** Severe procedure-coded inpatient events during inpatient treatment for head and neck cancer and thyroid cancer, 2005–2021 (result of the search in the DESTATIS database: *N* = 1,552,028 treatments for patients ≥ 30 years of age).

Inpatient Events	Number, Absolute * N	Number, Relative %
All inpatient treatments	1,552,028	100
Coded event		
No	1,252,942	80.73
Yes	299,086	19.27
All inpatient events	299,086	100
Cardiorespiratory/circulatory procedure-coded events, thrombosis	190,355	63.65
General symptoms	72,787	24.34
Impaired wound healing	16,195	5.41
Osteomyelitis, osteoradionecrosis	9414	3.15
Pneumothorax	5072	1.70
Reduced food intake	2910	0.97
Postoperative bleeding	1499	0.50
Diseases of the nose and paranasal sinuses	646	0.22
Speech, chewing, and swallowing disorders	184	0.06
Diseases of the ear	13	<0.01
Dental caries, periodontal problems, changes in the oral mucosa, abscesses	11	<0.01

* Absolute number of treatment episodes with the respective procedure-coded inpatient-event category.

**Table 5:** Multivariable analysis of factors associated with higher probability of severe procedure-coded inpatient events. The ordinal regression was calculated by DESTATIS using 1,552,028 observations, of which 1,551,955 were included in the final model. The 73 excluded observations had missing sex information.

Variable	β	SE	OR	CI	*p*
Intercept 4	−13.996	0.2586	0.00000083	0.00000050–0.00000139	<0.001
Intercept 3	−10.212	0.0414	0.0000367	0.0000339–0.0000398	<0.001
Intercept 2	−6.953	0.0160	0.00095	0.00093–0.00099	<0.001
Intercept 1	−3.4809	0.0135	0.0308	0.0300–0.0316	<0.001
Age	0.0025	0.0002	1.003	1.002–1.003	<0.001
Gender (Male)	−0.0734	0.0054	0.929	0.919–0.939	<0.001
C73 (Thyroid gland)	Reference		1		
C32 (Larynx)	0.9167	0.0098	2.501	2.453–2.550	<0.001
C10 (Oropharynx)	1.2035	0.0103	3.332	3.265–3.400	<0.001
C13 (Hypopharynx)	1.2242	0.0108	3.401	3.330–3.474	<0.001
C04 (Floor of the mouth)	1.5427	0.0104	4.677	4.583–4.774	<0.001
C09 (Tonsil)	0.9940	0.0119	2.702	2.640–2.766	<0.001
C02 (Unspecified part of the tongue)	1.3868	0.0112	4.002	3.915–4.091	<0.001
C01 (Base of the tongue)	1.1307	0.0126	3.098	3.022–3.175	<0.001
C07 (Parotid gland)	0.8937	0.0172	2.444	2.363–2.528	<0.001
C11 (Nasopharynx)	0.8696	0.0180	2.386	2.303–2.472	<0.001
C31 (Paranasal sinus)	1.1053	0.0167	3.020	2.923–3.121	<0.001
C05 (Palate)	1.1310	0.0161	3.099	3.002–3.198	<0.001
C03 (Gum)	1.7491	0.0149	5.749	5.584–5.920	<0.001
C30 (Nasal cavity and middle ear)	0.5369	0.0195	1.711	1.647–1.777	<0.001
C06 (Unspecified part of the mouth)	1.4930	0.0161	4.450	4.312–4.593	<0.001
C00 (Lip)	0.1915	0.0236	1.211	1.156–1.268	<0.001
C08 (Unspecified major salivary gland)	0.7817	0.0291	2.185	2.064–2.313	<0.001
C12 (Piriform sinus)	1.1412	0.0276	3.131	2.966–3.305	<0.001
C14 (Other sites lip, oral cavity, pharynx)	1.1850	0.0294	3.271	3.088–3.465	<0.001
C33 (Trachea)	1.6000	0.0322	4.953	4.650–5.276	<0.001
Surgery (Yes)	1.2427	0.0048	3.465	3.433–3.498	<0.001
Radiotherapy (Yes)	0.2921	0.0101	1.339	1.313–1.366	<0.001
Chemotherapy/immunotherapy (Yes)	3.3853	0.0110	29.527	28.897–30.170	<0.001
No CCI comorbidity	Reference		1		
1 CCI disease	0.4576	0.0051	1.580	1.565–1.596	<0.001
2 CCI diseases	0.6204	0.0079	1.860	1.831–1.889	<0.001
3 CCI diseases	0.6733	0.0137	1.961	1.909–2.014	<0.001
4 CCI diseases	0.7584	0.0259	2.135	2.029–2.246	<0.001
≥5 CCI diseases	0.7319	0.0547	2.079	1.868–2.314	<0.001

CCI = Charlson Comorbidity Index; β = regression coefficient; SE = standard error; OR = odds ratio; CI = 95% confidence interval.

## Data Availability

The underlying DESTATIS data cannot be made publicly available because they are subject to the confidentiality provisions governing official statistics in Germany. Access to the data is provided through the Research Data Centres of the Federal Statistical Office and the Statistical Offices of the Länder, subject to their applicable access procedures and requirements.

## Supplementary Materials

The following supporting information can be downloaded at: https://www.mdpi.com/article/10.3390/cancers18172860/s1, Supplementary Table S1: Primary tumor diagnosis: Included ICD-Codes. Supplementary Table S2: Therapies: Included OPS-Codes. Supplementary Table S3: Calculation of the Charlson Comorbidity Index: Included ICD-Codes. Supplementary Table S4: Procedure-coded inpatient event interventions: Included OPS-Codes. Supplementary Table S5: The various database queries and the resulting number of identified treatment cases. Supplementary Table S6: Primary head and neck cancer per age cohorts (Result of the search in the DESTATIS database: *N* = 1,499,781 treatments for patients ≥ 30 years of age and <90 years of age AND gender). The ICD and sex-specific values in this table are derived from the separate therapy query comprising 1,499,781 treatment episodes (see Supplementary Table S5) and therefore do not correspond to the full cohort of 1,552,028 treatment episodes. Supplementary Table S7: Charlson Comorbidity Index (CCI) per tumor subsite (Result of the search in the DESTATIS database: *N* = 1,552,028 treatments for patients ≥ 30 years of age). Supplementary Table S8: Therapy types and incidence rates per gender (Result of the search in the DESTATIS database: *N* = 572,017 treatments for patients ≥ 40 years of age). The therapy and sex-specific values in this table are derived from the separate therapy query comprising 572,017 treatment episodes (see Supplementary Table S5) and therefore do not correspond to the full cohort of 1,552,028 treatment episodes. Supplementary Table S9: RECORD statement. Supplementary Figure S1: Flowchart of the different data sets from each data query. Supplementary Figure S2: ICD-codes that led to hospitalization of head and neck cancer (HNC) patients in different age cohorts (color-coded) treated between 2005 and 2021 in German hospitals. (A): Men, (B): Women. X-axis: Absolute number of treatments, logarithmic scale. Y-axis: International Classification of Diseases (ICD). Supplementary Figure S3: Different types of therapy of head and neck cancer (HNC) patients in different age cohorts (color-coded) treated between 2005 and 2021 in German hospitals. (A): Men, (B): Women. X-axis: Absolute number of treatments, logarithmic scale. Reference [62] is cited in the Supplementary Materials.
