# Supplementary material for: The Impact of Comorbidity on Severe Procedure-Coded Inpatient Events in Head and Neck Cancer and Thyroid Cancer Hospitalizations in Germany: A Nationwide DRG Analysis, 2005–2021
Source: Cancers (Basel). 2026 Sep 4;18(17):2860. doi: 10.3390/cancers18172860 (PMC13565292; doi:10.3390/cancers18172860)
Supplement: Supplementary file 1 [file cancers-18-02860-s001.zip › cancers-4452016-supplementary.pdf]

Supplementary Tables

Supplementary Table S1. Primary tumor diagnosis: Included ICD-Codes.

ICD-Codes	Localization of the tumor
C00	Lip
C01	Base of the tongue
C02	Other and unspecified parts of tongue
C03	Gum
C04	Floor of the mouth
C05	Palate
C06	Other and unspecified parts of mouth
C07	Parotid gland
C08	Other and unspecified major salivary glands
C09	Tonsil
C10	Oropharynx
C11	Nasopharynx
C12	Piriform sinus
C13	Hypopharynx
C14	Other and ill-defined sites in the lip, oral cavity and pharynx
C30	Nasal cavity and middle ear
C31	Paranasal sinuses
C32	Larynx
C33	Trachea
C73	Thyroid gland

Supplementary Table S2. Therapies: Included OPS-Codes.

Therapy	OPS-Codes
Surgery (OPS-code search only with three-digit numbers and asterisk (*) as placeholder)	5-06*, 5-08* - 5-16*, 5-18* - 5-20*, 5-21* - 5-23* - 5-28* - 5-31*, 5-40*, 5-76*, 5-77*, 5-89*
Radiotherapy	8-520.0, 8-520.1, 8-521.0, 8-521.1, 8-522.0, 8-522.1, 8-522.2, 8-522.3, 8-522.6, 8-522.9, 8-523.0, 8-523.1, 8-523.2, 8-523.3, 8-523.4, 8-523.5, 8-523.6, 8-523.7, 8-524.3, 8-524.5, 8-524.6, 8-525.0, 8-525.1, 8-525.2, 8-

	525.3, 8-525.4, 8-526.0, 8-526.1, 8-526.2, 8-528.6, 8-528.7, 8-528.8, 8-528.9, 8-529.3, 8-529.4, 8-529.5, 8-529.6, 8-529.7, 8-529.8
Chemotherapy, Immunotherapy	8-541.0, 8-541.1, 8-541.2, 8-541.3, 8-541.4, 8-541.5, 8-541.6, 8-542.1, 8-543.1, 8-543.2, 8-543.3, 8-543.4, 8-543.5, 8-543.6, 8-543.7, 8-543.8, 8-543.9, 8-546.0, 8-546.1, 8-547.0, 8-547.1, 8-547.2, 8-547.3, 8-548.0, 8-548.1, 8-549.0

Supplementary Table S3. Calculation of the Charlson Comorbidity Index: Included ICD-Codes.

Disease (d)	Points	ICD-Codes
Heart attack (d1)	1	I21, I22
Heart failure (d2)	1	I50
Peripheral arterial disease (d3)	1	I70–I74, I77
Cerebrovascular disease (d4)	1	G45, I60–I69
Dementia (d5)	1	F00–F03
Chronic lung disease (d6)	1	J40–J47, J60–J67, J68.4, J70.1, J70.3
Collagenosis / connective tissue disease (d7)	1	M05–M06, M08–M09, M12–M13, M30–M36
Peptic ulcer disease (d8)	1	K25–K28
Mild liver disease (d9)**	1	B18; K70.0–K70.3, K70.9; K71.3–K71.5, K71.7; K73; K74; K75; K76.0–K76.5, K76.8–K76.9
Diabetes mellitus without chronic complications (d10)***	1	E10.0, E10.1, E10.6, E10.8, E10.9; E11.0, E11.1, E11.6, E11.8, E11.9; E12.0, E12.1, E12.6, E12.8, E12.9; E13.0, E13.1, E13.6, E13.8, E13.9; E14.0, E14.1, E14.6, E14.8, E14.9
Hemiplegia	2	G81–G82
Moderate and severe kidney disease (d11)	2	N17–N19
Diabetes mellitus with chronic complications (d12)***	2	E10.2–E10.5, E10.7; E11.2–E11.5, E11.7; E12.2–E12.5, E12.7; E13.2–E13.5, E13.7; E14.2–E14.5, E14.7
Cancer*	2	C00–C97
Leukemia (d13)	2	C91–C95
Lymphoma (d14)	2	C81–C85, C88, C90, C96

Moderate and severe liver disease (d15)**	3	I85.0, I85.9, I86.4, I98.2; K70.4; K71.1; K72.1, K72.9; K76.5–K76.7
Metastatic solid tumor (d16)	6	C77–C80
Acquired Immune Deficiency Syndrome (AIDS)	6	B20–B24

*only counted if the ICD code was different from the ICD code for the primary cancer to avoid double counting of ICD codes

**if mild liver disease (d9) AND moderate and severe liver disease (d15) was coded, only severe liver disease was counted. Only the severest liver disease code was counted to avoid double counting of ICD codes.

***if Diabetes mellitus (without end-organ damage) (d10) AND diabetes mellitus with end-organ damage (d12) was coded, only diabetes mellitus with end-organ damage was counted. Only the severest liver diabetes mellitus code was counted to avoid double counting of ICD codes

d1: Patients hospitalized due to a myocardial infarction confirmed by electrocardiogram and/or enzyme tests

d2: Patients with nocturnal or exertional dyspnea whose symptoms improve with treatment

d3: Patients with intermittent claudication, following peripheral bypass surgery, with acute arterial occlusion or gangrene, as well as those with untreated abdominal or thoracic aortic aneurysms >6 cm

d4: Patients with transient ischemic attack (TIA) or stroke without severe residual deficits

d5: Patients with chronic cognitive impairment

d6: Patients with pulmonary dyspnea during light or moderate exertion without treatment, or patients with paroxysmal dyspnea (asthma)

d7: Patients with polymyalgia rheumatica, systemic lupus erythematosus, severe rheumatoid arthritis, polymyositis

d8: Patients who have previously been treated for ulcers

d9: Patients with liver cirrhosis without portal hypertension

d10: Patients with diabetes mellitus receiving medication

d11: Patients with requirement for dialysis or creatinine >3 mg/dL; or previous hospitalizations due to hyperosmolar coma or ketoacidosis

d12: Patients with any other solid cancers without evidence of metastasis within the last five years

d13: Patients with acute and chronic leukemias

d14: Patients with Hodgkin's and non-Hodgkin's lymphomas, multiple myeloma

d15: Patients with liver cirrhosis with portal hypertension without a history of bleeding and patients with a history of variceal bleeding

d16: Any other tumor, not of the primary head and neck cancer

* The Cancer category comprises malignant neoplasms (C00–C97). Where a diagnosis meets criteria for a more specific CCI category (e.g., leukemia, lymphoma, or metastatic solid tumor), the corresponding specific category is assigned according to the hierarchical CCI algorithm.

** Liver disease is separated into mild liver disease (1 point) and moderate/severe liver disease (3 points) using fourth-digit-resolved ICD-10 definitions from established ICD-10 adaptations of the Charlson Comorbidity Index. The distinction is based on the specificity of the ICD-10 code rather than on clinical adjudication.

*** Diabetes mellitus is separated into diabetes without chronic complications (1 point) and diabetes with chronic complications (2 points) using fourth-digit-resolved ICD-10 definitions from established ICD-10 adaptations of the Charlson Comorbidity Index.

Supplementary Table S4. Procedure-coded inpatient event interventions: Included OPS-Codes.*

**Potential complications related to the procedure-coded inpatient event**	**Interventions**	**OPS-Codes included if this coding was performed in addition to the OPS coding for the therapy of the primary disease AND if secondary ICD codes were coded for case closure at demission in addition to the ICD diagnosis coding at the time of admission.**
General symptoms	Administration of medications<br>Administration of medications and nutrition<br>Therapeutic injection	all 6-00 codes<br>8-011, 8-019<br><br>8-020
Difficulty eating	Administration of medications and nutrition	8-015, 8-016, 8-017, 8-018
Bleeding, shock	Transfusion of blood cells<br><br><br><br><br>Collection and transfusion of autologous blood<br><br>Transfusion of plasma, plasma components, and infusion of volume expanders	8-800.0, 8-800.1, 8-800.6, 8-800.c, 8-800.d, 8-800.f, 8-800.g, 8-800.h, 8-800.k, 8-800.m, 8-800.n, 8-800.p, 8-801, 8-802, 8-803<br>8-810, 8-810.6, 8-810.7, 8-810.8, 8-810.9, 8-810.a, 8-810.b<br>8-810.c, 8-810.d, 8-810.e, 8-810.g, 8-810.h, 8-810.j, 8-810.q, 8-810.s, 8-810.t, 8-810.w, 8-812
Cardiorespiratory/circulatory procedure-coded events, thrombosis	Resuscitation procedures<br>Monitoring of respiration, heart, and circulation	8-771, 8-772, 8-779<br>8-930, 8-931, 8-9310, 8-9311, 8-932, 8-933

	Therapeutic catheterization and insertion of cannulas into vessels<br>Procedures for the respiratory system<br><br>Systemic thrombolysis<br>Thrombin injection<br>Thrombolysis	8-8310, 8-835, 8-836, 8-837, 8-838, 8-839, 8-700, 8-7000, 8-7001, 8-701, 8-704, 8-706, 8-713, 8-714<br>8-020.8<br>8-020.c<br>8-020.d
Wound healing disorders, burns, chemical burns	Dressings and removal of diseased tissue from the skin and subcutaneous tissue<br>Surgeries on the skin and subcutaneous tissue<br>Surgery on the skin and subcutaneous tissue for burns and chemical burns	8-190, 8-191, 8-192<br><br><br>5-892, -5894, 5-985, 5-896, 5-900<br><br>5-920, 5-921, 5-922, 5-923, 5-924, 5-925, 5-926, 5-927, 5-928, 5-929
Osteomyelitis, osteoradionecrosis	Other surgery on the facial skull bones	5-770, 5-7700, 5-77010, 5-77011, 5-77012, 5-7704, 5-7705, 5-7707, 5-771, 5-772
Dental caries, periodontal problems, changes in the oral mucosa, abscesses	Extraction and restoration of teeth<br><br>Surgery on the gums, alveoli, and jaw<br>Surgery on the tongue<br>Surgery on the salivary glands and ducts<br>Other surgery on the mouth and face	5-230, 5-231, 5-232, 5-233, 5-235, 5-236, 5-237<br>5-240, 5-241, 5-242, 5-243, 5-244, 5-249<br>5-250, 5-251, 5-252, 5-253, 5-259<br>5-260, 5-261, 5-262, 5-263, 5-269, 5-270, -5271, 5-273, 5-274, 5-275, 5-277, 5-278, 5-279
Diseases of the nose and paranasal sinuses	Surgery on the nose<br><br><br>Paranasal sinus surgery	5-210, 5-211, 5-212, 5-213, 5-214, 5-215, 5-216, 5-217, 5-218, 5-219<br>5-220, 5-221, 5-222, 5-223, 5-224, 5-225, 5-229

Pneumothorax	Surgery on the chest wall, mediastinum, and diaphragm<br>Therapeutic aspiration and drainage by puncture	5-340.0<br><br>8-152.1
Diseases of the ear	Ear surgery	5-180, 5-181, 5-182, 5-183, 5-185, 5-187, 5-188, 5-189, 5-19, 5-20
Speech, chewing, and swallowing disorders	Comprehensive early rehabilitation treatment for patients with head and neck cancer	8-553*, 8-553.0, 8-553.1, 8-553.2, 8-553.3, 8-553.4, 8-553.5, 8-553.6

*If the search was not performed using the five-digit OPS code, but only a three- or four-digit code, then the search was performed using an asterisk as a placeholder after the code.

Supplementary Table S5. The various database queries and the resulting number of identified treatment cases.

Query	Search criterion	Purpose	Number of included treatments cases, absolute *N*
1	ICD codes for head and neck cancer, thyroid cancer **AND** ≥30 years of age **AND** OPS-codes for severe inpatient events.	Overall cohort	1,552,028
2	ICD codes for head and neck cancer, thyroid cancer AND ⩾30 years of age AND <90 years of age AND OPS-codes for severe inpatient events AND gender.	Overall cohort <90 years of age	1,499,781
3	ICD codes for head and neck cancer, thyroid cancer **AND** ≥30 years of age **AND** OPS-codes for severe inpatient events **AND** gender	Sex-stratified analysis	1,228,215
4	ICD codes head and neck cancer, thyroid cancer **AND** ≥30 years of age **AND** OPS-codes for severe inpatient events **AND** ICD-codes for CCI **AND** gender	CCI + sex analysis	1,222,367
5	ICD codes head and neck cancer, thyroid cancer **AND** ≥40 years of age **AND** OPS-codes for severe inpatient events **AND** gender **AND** OPS-codes for the treatment of the primary cancer	Treatment-modality analysis	572,017

6	age, sex, localization, operation, radiotherapy, chemotherapy, CCI, event count	ordinal regression	1,551,955

CCI = Charlson Comorbidity Index

Supplementary Table S6. Primary head and neck cancer per age cohorts (Result of the search in the DESTATIS database: *N* = 1,499,781 treatments for patients ≥ 30 years of age and <90 years of age AND gender). The ICD and sex-specific values in this table are derived from the separate therapy query comprising 1,499,781 treatment episodes (see **Supplementary Table S5**) and therefore do not correspond to the full cohort of 1,552,028 treatment episodes.

**Men**						
**Age (years)**	**30-39**	**40-49**	**50-59**	**60-69**	**70-79**	**80-89**
all	20,167	101,327	301,740	340,512	203,105	51,161
C00	137	634	1,776	3,575	6,653	3,929
C01	446	4,724	18,336	20,785	10,750	2,024
C02	1,537	6,555	18,427	19,580	11,003	2,621
C03	211	1,461	4,529	5,480	4,191	1,484
C04	761	10,842	32,531	29,156	11,597	1,958
C05	283	1,947	6,940	7,266	3,515	833
C06	241	1,487	4,334	4,829	3,608	1,288
C07	568	1,618	3,421	4,670	6,467	3,981
C08	160	634	1,316	1,485	1,441	499
C09	437	7,331	24,120	24,663	11,467	2,103
C10	669	11,915	45,308	47,817	20,479	3,390
C11	1,467	3,932	7,236	6,802	3,724	646
C12	20	738	2,641	3,294	1,765	329
C13	482	10,387	40,417	45,327	22,352	3,456
C14	48	664	2,279	2,479	1,196	225
C30	584	2,178	4,463	4,946	3,893	1,242
C31	692	2,604	5,540	5,792	4,990	1,435
C32	958	12,666	53,520	78,783	56,342	14,905
C33	84	270	938	1,450	1,143	309
C73	10,353	18,740	23,668	22,333	16,529	4,504
**Women**						
all	36,053	70,758	120,874	126,536	90,811	36,737
C00	64	299	680	1,606	3,504	2,968
C01	179	1,365	5,613	6,132	3,697	1,085
C02	1,028	2,753	6,972	8,475	7,293	3,537
C03	86	563	2,043	3,488	4,117	2,613
C04	231	2,235	8,137	9,424	5,725	2,053
C05	201	858	2,596	3,325	2,358	1,032
C06	177	655	2,029	3,089	3,341	2,009
C07	628	1,277	2,095	2,586	3,269	2,247
C08	176	519	885	983	1,049	605
C09	210	2,122	7,451	8,432	4,380	1,194
C10	176	2,663	11,486	12,924	6,371	1,492
C11	718	1,790	2,173	2,148	1,347	475
C12	0	57	404	506	227	63

C13	118	1,498	6,385	7,768	3,263	740
C14	12	133	530	637	392	177
C30	428	1,519	2,804	3,019	2,469	1,140
C31	383	1,108	2,052	2,532	2,545	1,339
C32	362	2,482	9,669	11,927	7,388	2,069
C33	41	226	418	684	635	188
C73	30,799	46,610	46,452	36,851	27,441	9,688

Supplementary Table S7. Charlson comorbidity index (CCI) per tumor subsite (Result of the search in the DESTATIS database: N = 1,552,028 treatments for patients ≥ 30 years of age).

ICD code	Tumor subsite	Mean CCI	Number, absolute *N*	Number, relative %
all		1.026	1,552,028	100
C14	Other sites in the lip, oral cavity and pharynx	1.466	8,904	0.57
C12	Piriform sinus	1.462	10,084	0.65
C13	Hypopharynx	1.458	142,589	9.19
C09	Tonsil	1.394	94,305	6.08
C08	Other and unspecified major salivary glands	1.393	10,194	0.66
C10	Oropharynx	1.392	165,253	10.65
C01	Base of the tongue	1.330	75,641	4.87
C11	Nasopharynx	1.244	34,692	2.24
C07	Parotid gland	1.238	35,344	2.28
C33	Trachea	1.207	6,565	0.42
C04	Floor of the mouth	1.188	115,375	7.43
C05	Palate	1.160	31,829	2.05
C06	Other and unspecified parts of mouth	1.137	27,883	1.80
C02	Other and unspecified parts of tongue	1.097	91,896	5.92
C03	Gum	1.003	31,103	2.00
C31	Paranasal sinuses	0.931	32,149	2.07
C00	Lip	0.877	27,471	1.77
C32	Larynx	0.811	253,082	16.31
C30	Nasal cavity and middle ear	0.704	29,783	1.92
C73	Thyroid gland	0.514	327,886	21.13

Supplementary Table S8. Therapy types and incidence rates per gender (Result of the search in the DESTATIS database: N = 572,017 treatments for patients ≥ 40 years of age). The therapy and sex-specific values in this table are derived from the separate therapy query comprising 572,017 treatment episodes (see **Supplementary Table S5**) and therefore do not correspond to the full cohort of 1,552,028 treatment episodes.

	**All**			**Men**			**Women**		
	**Abs.**	**Rel.**	**IR**	**Abs.**	**Rel.**	**IR**	**Abs.**	**Rel.**	**IR**
	n	%		*n*	%		*n*	%	
All therapies	572,017	100	72.18	394,917	100	104.88	177,100	100	42.57
Surgery	455,346	79.60	57.45	303,831	76.94	80.69	151,515	85.55	36.42
Radiotherapy	64,541	11.28	8.14	49,649	12.57	13.19	14,892	8.41	3.58
Chemotherapy/Immunotherapy	41,900	7.32	5.29	33,569	8.50	8.92	8,331	4.70	2.00
Radiochemotherapy	2,238	0.39	0.28	1,758	0.45	0.47	480	0.27	0.12

Surgery and chemotherapy	906	0.16	0.11	719	0.18	0.19	187	0.11	0.04
Surgery and radiotherapy	6,979	1.22	0.88	5,311	1.34	1.41	1,668	0.94	0.40
Surgery and radiochemotherapy	107	0.02	0.01	80	0.02	0.02	27	0.02	0.01

IR = incidence rate per age cohort per 100,000 population related to the German population 40+ years of age; abs. = absolute; rel. = relative

Supplementary Table S9. RECORD statement
for
Müller-Anderski et al. The Impact of Comorbidity on Severe Procedure-Coded Inpatient Events in Head and Neck Cancer and Thyroid Cancer Hospitalizations in Germany: A Nationwide DRG Analysis, 2005-2021

	Item No.	STROBE items	Location in manuscript where items are reported	RECORD items	Location in manuscript where items are reported
**Title and abstract**					
	1	(a) Indicate the study's design with a commonly used term in the title or the abstract (b) Provide in the abstract an informative and balanced summary of what was done and what was found	Page 1, 2	RECORD 1.1: The type of data used should be specified in the title or abstract. When possible, the name of the databases used should be included.<br><br>RECORD 1.2: If applicable, the geographic region and timeframe within which the study took place should be reported in the title or abstract.<br><br>RECORD 1.3: If linkage between databases was conducted for the study, this should be clearly stated in the title or abstract.	Page 1, 2<br><br>Page 1, region: Germany, 2005 to 2021 in title and abstract<br><br>Not applicable
**Introduction**					
Background rationale	2	Explain the scientific background and rationale for the investigation being reported	Page 2, 3		
Objectives	3	State specific objectives, including any prespecified hypotheses	Page 3		
**Methods**					
Study Design	4	Present key elements of study design early in the paper	Page 3, 4		

Setting	5	Describe the setting, locations, and relevant dates, including periods of recruitment, exposure, follow-up, and data collection	Page 3, 4 (no follow-up)		
Participants	6	(a) Cohort study - Give the eligibility criteria, and the sources and methods of selection of participants. Describe methods of follow-up<br>Case-control study - Give the eligibility criteria, and the sources and methods of case ascertainment and control selection. Give the rationale for the choice of cases and controls<br>Cross-sectional study - Give the eligibility criteria, and the sources and methods of selection of participants<br><br>(b) Cohort study - For matched studies, give matching criteria and number of exposed and unexposed<br>Case-control study - For matched studies, give matching criteria and the number of controls per case	Selection of ICD-codes and OPS-codes in the Methods, page 3-4 and in the Supplement Tables	RECORD 6.1: The methods of study population selection (such as codes or algorithms used to identify subjects) should be listed in detail. If this is not possible, an explanation should be provided.<br><br>RECORD 6.2: Any validation studies of the codes or algorithms used to select the population should be referenced. If validation was conducted for this study and not published elsewhere, detailed methods and results should be provided.<br><br>RECORD 6.3: If the study involved linkage of databases, consider use of a flow diagram or other graphical display to demonstrate the data linkage process, including the number of individuals with linked data at each stage.	ICD-codes and OPS-codes completely listed as used for the data queries.<br><br>No formal validation<br><br>No linkage
Variables	7	Clearly define all outcomes, exposures, predictors, potential confounders, and effect modifiers. Give diagnostic criteria, if applicable.	Not applicable	RECORD 7.1: A complete list of codes and algorithms used to classify exposures, outcomes, confounders, and effect modifiers should be provided. If these cannot be reported, an explanation should be provided.	All codes are listed in the Supplementary Tables
Data sources/ measurement	8	For each variable of interest, give sources of data and details of methods of assessment (measurement).<br>Describe comparability of assessment methods if	The data source is DESTATIS for all all datasets, this is clearly indicated on page 3		

		there is more than one group			
Bias	9	Describe any efforts to address potential sources of bias	Not applicable, health care data set analysis		
Study size	10	Explain how the study size was arrived at	Not applicable		
Quantitative variables	11	Explain how quantitative variables were handled in the analyses. If applicable, describe which groupings were chosen, and why	Descriptive analyses mainly, groupings of codings necessary because of data protection regulations		
Statistical methods	12	(a) Describe all statistical methods, including those used to control for confounding<br>(b) Describe any methods used to examine subgroups and interactions<br>(c) Explain how missing data were addressed<br>(d) Cohort study - If applicable, explain how loss to follow-up was addressed<br>Case-control study - If applicable, explain how matching of cases and controls was addressed<br>Cross-sectional study - If applicable, describe analytical methods taking account of sampling strategy<br>(e) Describe any sensitivity analyses	Page 4, 5		
Data access and cleaning methods			Page 2, 3	RECORD 12.1: Authors should describe the extent to which the investigators had access to the database population used to create the study population.<br><br>RECORD 12.2: Authors should provide information on the	Standardized process for data queries using DESTATIS DRG data, page 3

				data cleaning methods used in the study.	Data delivered by DESTATIS AFTER data cleaning
Linkage		..	No linkage performed	RECORD 12.3: State whether the study included person-level, institutional-level, or other data linkage across two or more databases. The methods of linkage and methods of linkage quality evaluation should be provided.	No linkage,<br><br>Data on treatment level, not on person-level, page 3
**Results**					
Participants	13	(a) Report the numbers of individuals at each stage of the study (e.g., numbers potentially eligible, examined for eligibility, confirmed eligible, included in the study, completing follow-up, and analysed)<br>(b) Give reasons for non-participation at each stage.<br>(c) Consider use of a flow diagram	Page 5 and tables	RECORD 13.1: Describe in detail the selection of the persons included in the study (i.e., study population selection) including filtering based on data quality, data availability and linkage. The selection of included persons can be described in the text and/or by means of the study flow diagram.	Selection by ICD and OPS-codes, all listed in Supplement Tables<br><br>Study flow diagram is part of the supplements: Supplement Figure S1
Descriptive data	14	(a) Give characteristics of study participants (e.g., demographic, clinical, social) and information on exposures and potential confounders<br>(b) Indicate the number of participants with missing data for each variable of interest<br>(c) Cohort study - summarise follow-up time (e.g., average and total amount)	Page 5, tables, supplement tables; no information on confounders		
Outcome data	15	Cohort study - Report numbers of outcome events or summary measures over time<br>Case-control study - Report numbers in each exposure category, or	Results, page 5 – 12; Tables 1-5 and Figures 1-2 as well as supplements		

		summary measures of exposure<br>Cross-sectional study - Report numbers of outcome events or summary measures			
Main results	16	(a) Give unadjusted estimates and, if applicable, confounder-adjusted estimates and their precision (e.g., 95% confidence interval). Make clear which confounders were adjusted for and why they were included<br>(b) Report category boundaries when continuous variables were categorized<br>(c) If relevant, consider translating estimates of relative risk into absolute risk for a meaningful time period	Mainly simple descriptive data presented. Multivariate analysis (Table 5) with confidence intervals		
Other analyses	17	Report other analyses done—e.g., analyses of subgroups and interactions, and sensitivity analyses	Not applicable		
**Discussion**					
Key results	18	Summarise key results with reference to study objectives	Page 12, first paragraph		
Limitations	19	Discuss limitations of the study, taking into account sources of potential bias or imprecision. Discuss both direction and magnitude of any potential bias	Limitations section on page 14	RECORD 19.1: Discuss the implications of using data that were not created or collected to answer the specific research question(s). Include discussion of misclassification bias, unmeasured confounding, missing data, and changing eligibility over time, as they pertain to the study being reported.	In detail discussed on page 14
Interpretatio n	20	Give a cautious overall interpretation of results considering objectives, limitations, multiplicity of analyses, results from	Page 13 - 15		

		similar studies, and other relevant evidence			
Generalisability	21	Discuss the generalisability (external validity) of the study results	Page 13- 15, German data		
**Other Information**					
Funding	22	Give the source of funding and the role of the funders for the present study and, if applicable, for the original study on which the present article is based	Page 15		
Accessibility of protocol, raw data, and programming code		..	Page 15	RECORD 22.1: Authors should provide information on how to access any supplemental information such as the study protocol, raw data, or programming code.	Large set of Supplement Material, and page 15

*Reference: Benchimol EI, Smeeth L, Guttmann A, Harron K, Moher D, Petersen I, Sørensen HT, von Elm E, Langan SM, the RECORD Working Committee. The REporting of studies Conducted using Observational Routinely-collected health Data (RECORD) Statement. PLoS Medicine 2015; in press.

*Checklist is protected under Creative Commons Attribution (CC BY) license.

Supplementary Figures

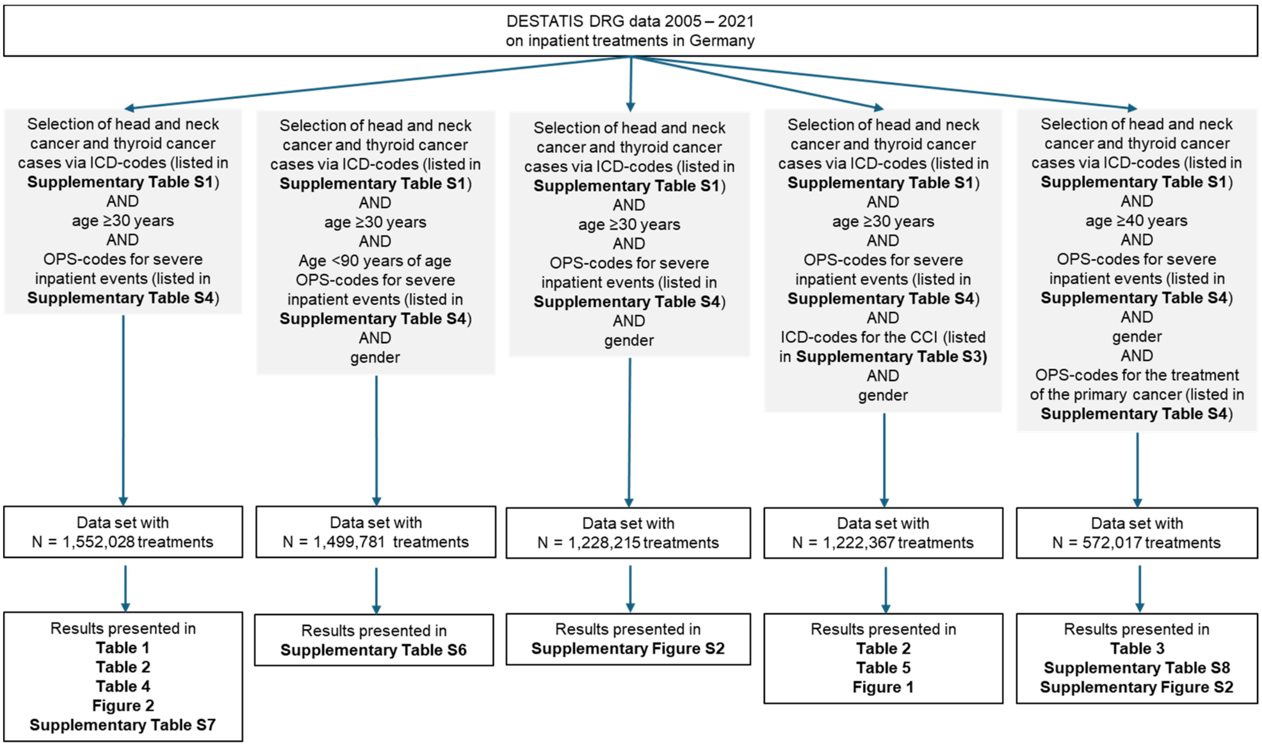

Supplementary Figure S1. Flowchart of the different data sets from each data query.

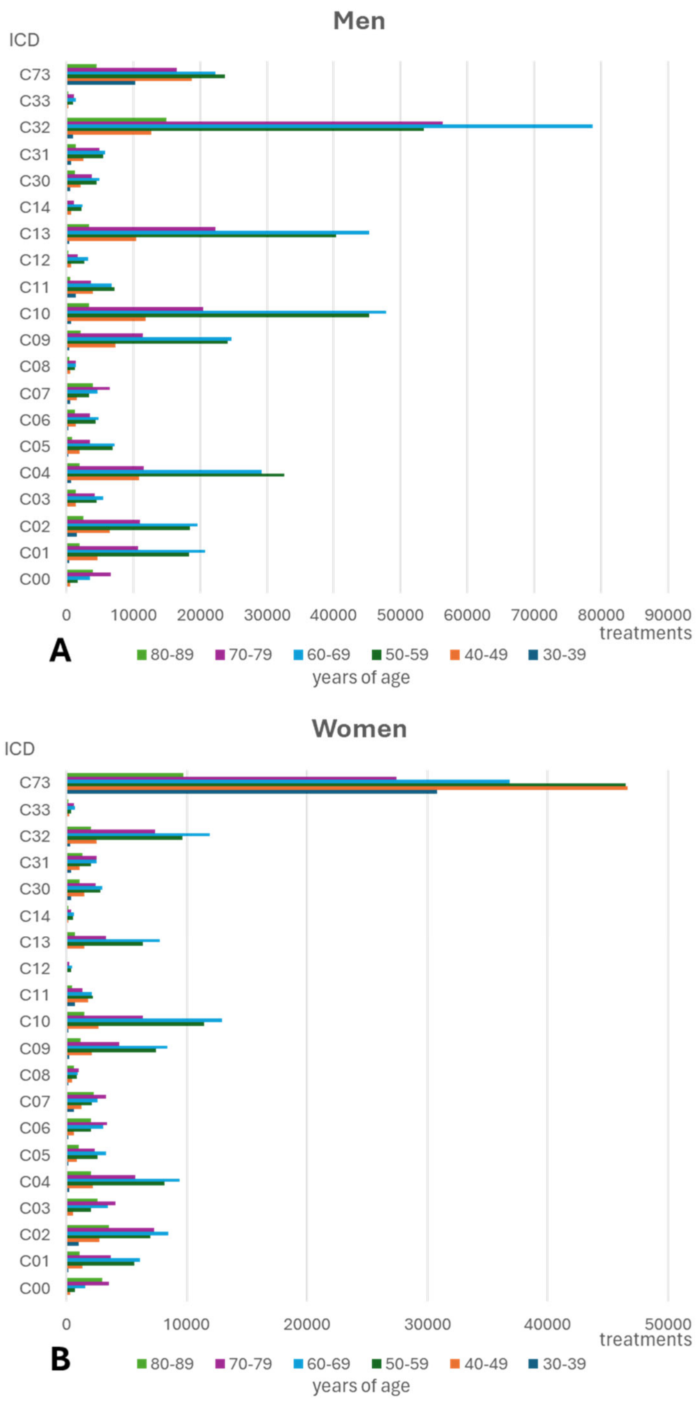

Supplementary Figure S2. ICD codes that led to hospitalization of head and neck cancer (HNC) patients in different age cohorts (color-coded) treated between 2005 and 2021 in German hospitals. (**A**): Men, (**B**): Women. X-axis: Absolute number of treatments, logarithmic scale. Y-axis: International Classification of Diseases (ICD).

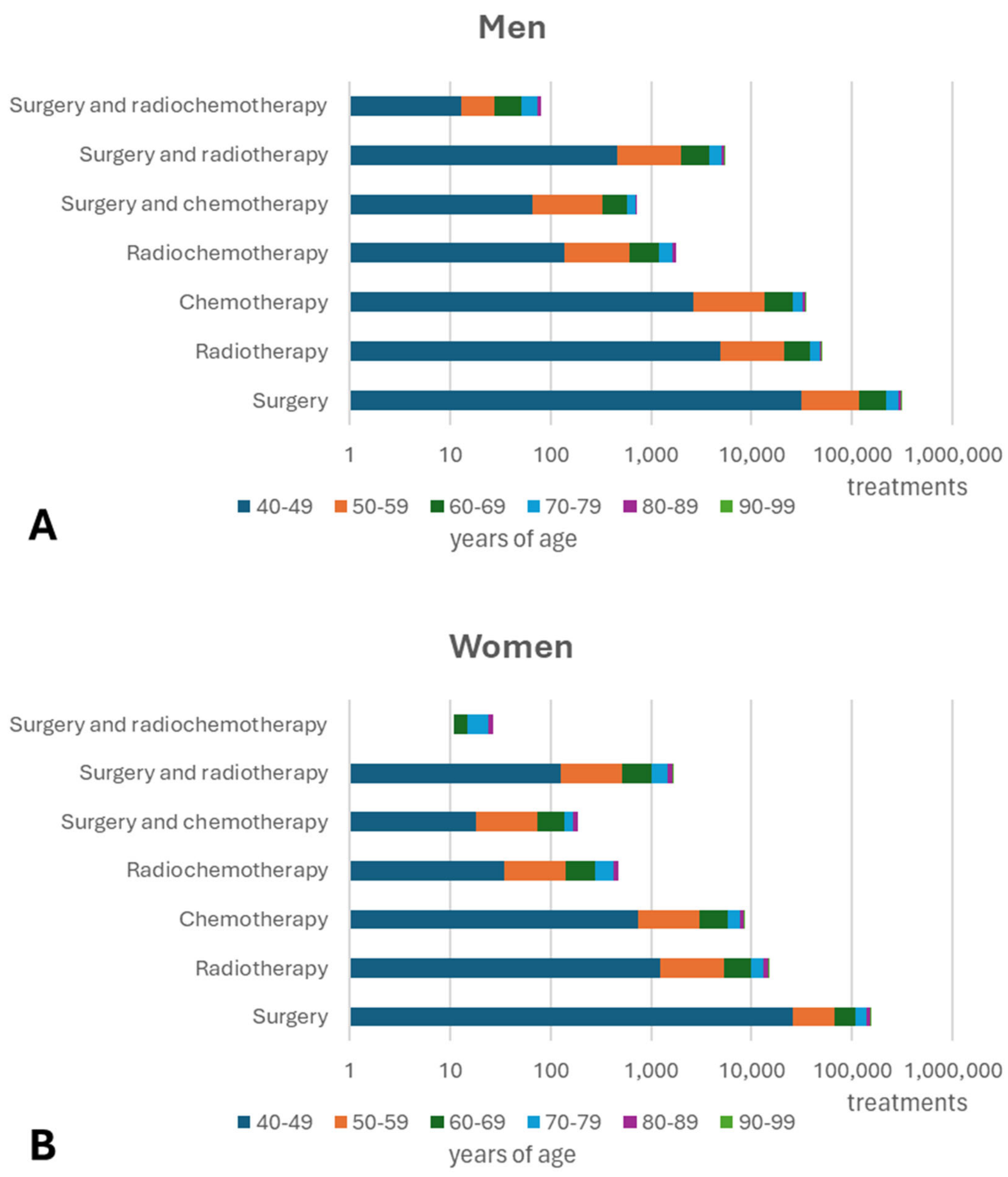

Supplementary Figure S3. Different types of therapy of head and neck cancer (HNC) patients in different age cohorts (color-coded) treated between 2005 and 2021 in German hospitals. (**A**): Men, (**B**): Women. X-axis: Absolute number of treatments, logarithmic scale.
